# Modelling dynamic change of malaria transmission in holoendemic setting (Dielmo, Senegal) using longitudinal measures of antibody prevalence to *Plasmodium falciparum* crude schizonts extract

**DOI:** 10.1186/s12936-017-2052-0

**Published:** 2017-10-11

**Authors:** Oumy Niass, Philippe Saint-Pierre, Makhtar Niang, Fode Diop, Babacar Diouf, Michel Matar Faye, Fatoumata Diène Sarr, Joseph Faye, Nafissatou Diagne, Cheikh Sokhna, Jean-François Trape, Ronald Perraut, Adama Tall, Abdou Kâ Diongue, Aïssatou Toure Balde

**Affiliations:** 1 Immunology Unit, Institut Pasteur Dakar, 36 Avenue Pasteur, BP 220, Dakar, Senegal; 2 Laboratoire d’étude et de Recherche en Statistique et Développement, Université Gaston Berger, BP 237, Saint-Louis, Senegal; 3 Institut Mathématique de Toulouse, Université Paul Sabatier, Toulouse, France; 4 Epidemiology of Infectious Diseases Unit, Institut Pasteur Dakar, 36 Avenue Pasteur, BP 220, Dakar, Senegal; 5 Institut de Recherche pour le Développement, BP 1386, Dakar, Senegal

## Abstract

**Background:**

Evaluation of local *Plasmodium falciparum* malaria transmission has been investigated previously using the reversible catalytic model based on prevalence of antibody responses to single antigen to estimate seroconversion rates. High correlations were observed between seroconversion rates and entomological inoculation rates (EIR). However, in this model, the effects of malaria control interventions and clinical episodes on serological measurements were not assessed. This study monitors the use of antibody responses to *P. falciparum* crude extracts for assessing malaria transmission, compares seroconversion rates estimated from longitudinal data to those derived from cross-sectional surveys and investigates the effects of malaria control interventions on these measures in an area of declining malaria transmission. In addition, the validity of this model was evaluated by comparison with the alternative model.

**Methods:**

Five cross-sectional surveys were carried out at the end of the wet season in Dielmo, a malaria-endemic Senegalese rural area in 2000, 2002, 2008, 2010 and 2012. Antibodies against schizonts crude extract of a local *P. falciparum* strain adapted to culture (*Pf 07/03*) were measured by ELISA. Age-specific seroprevalence model was used both for cross-sectional surveys and longitudinal data (combined data of all surveys).

**Results:**

A total of 1504 plasma samples obtained through several years follow-up of 350 subjects was used in this study. Seroconversion rates based on *P. falciparum* schizonts crude extract were estimated for each cross-sectional survey and were found strongly correlated with EIR. High variability between SCRs from cross-sectional and longitudinal surveys was observed. In longitudinal studies, the alternative catalytic reversible model adjusted better with serological data than the catalytic model. Clinical malaria attacks and malaria control interventions were found to have significant effect on seroconversion.

**Discussion:**

The results of the study suggested that crude extract was a good serological tool that could be used to assess the level of malaria exposure in areas where malaria transmission is declining. However, additional parameters such as clinical malaria and malaria control interventions must be taken into account for determining serological measurements for more accuracy in transmission assessment.

**Electronic supplementary material:**

The online version of this article (doi:10.1186/s12936-017-2052-0) contains supplementary material, which is available to authorized users.

## Background

Falciparum malaria remains a public health priority and a major cause of morbidity and mortality in tropical areas [1]. The observed pathophysiology of *Plasmodium falciparum* malaria infection is strongly dependent upon endemicity, age and level of immunity [2]. In malaria endemic areas, where transmission is perennial and stable, parasitic tolerance has been described and explained by a partial immunity acquired over years [3, 4]. Age is, therefore, a major indicator of the duration of exposure to malaria parasite. However, acquired immunity is not completely protective but can be effective against clinical symptoms and severe form of the disease [5].

Several interventions have been implemented during the two last decades in the fight against malaria. These interventions include treatment by artemisinin-based combination therapy (ACT), use of long-lasting insecticide-impregnated bed nets for exposed populations and use of rapid diagnostic tests for malaria diagnosis. These interventions led to considerable reduction in the number of clinical episodes and deaths due to malaria [1]. Despite the significant progress achieved, the disease remains a major problem. The World Health Organization (WHO) reported 212 million clinical malaria cases and 429,000 deaths in 2015. Children under 5 years old are particularly susceptible to malaria illness, infection and death. In 2015, malaria killed an estimated 303,000 under-fives globally, including 292,000 in the African Region [1].

Monitoring malaria transmission intensity and strengthening malaria control measures are necessary since incidence of severe disease and mortality increase with transmission intensity [6–8]. The ongoing changes in malaria endemicity become a key determinant of the progress achieved. Moreover these changes determine the time required to reach the step where elimination could be foreseen [9]. Standard measurements of malaria transmission based on entomological inoculation rate (EIR) and parasite prevalence are expensive, time-consuming and lack of precision because of micro-heterogeneity of malaria transmission [10–12]. In addition, both EIR and parasite prevalence are affected by seasonality [13, 14]. Furthermore, assessing malaria transmission intensity and evaluating the impact of interventions are complicated in areas where transmission has been substantially reduced. Therefore, alternative approaches are required to assess malaria transmission and evaluate intervention programmes.

The use of mathematical models and prevalence of anti-malarial antibodies constitute alternative approaches to evaluate malaria endemicity [15, 16]. Mathematical models allow the determination of seroconversion rate (SCR) and seroreversion rate (SRR) which are, for a given time interval, the rates with which a seronegative subject becomes seropositive and a seropositive subject turns back to seronegative, respectively. Predictive seroprevalence (prevalence of antibody responses) can be calculated by using maximum likelihood methods. Seroprevalence reflects cumulative exposure and thus is less affected by seasonality because antibody responses can persist for years after infection. Serological markers have been previously used to assess malaria transmission intensity [15–17], to detect recent changes in malaria endemicity [17, 18], to evaluate effectiveness of malaria eradication efforts [19, 20], and SCR has been shown to correlate with EIR [15]. However, these single antigen-based serological approaches have significant limitations related to the sensitivity of the assay and the antigenic polymorphism that may affect detectable antibody responses [21–23].

An alternative method could be the use of a combination of antigens rather than a single one since recent studies have shown that responses to crude parasite extract or multiple antigens can overcome [17, 24, 25] these limitations.

Almost all reported mathematical approaches used the catalytic model without considering the effect of several factors (covariates). Taking these covariates into account could improve the effectiveness of serological models in assessing the intensity of malaria transmission [15, 16, 18, 25–29].

Incorporation of covariates such as the number of clinical attack or the use of long-lasting insecticide-impregnated bed nets (LLIN) in the model, allows to take into account the heterogeneity of the population and the effect of interventions affecting malaria transmission.

Previous studies have estimated SCR with reversible catalytic model using cross-sectional surveys and high correlation was shown between serological measurements of transmission and EIR [15, 16, 18]. However, given the fact that longitudinal surveys are often considered in sero-epidemiological studies and the increasing use of cross-sectional serological data to investigate malaria transmission, it would be important to assess consistency of cross-sectional results with SCR estimated from longitudinal survey in order to validate this approach. In this context, Arnold et al. [30] investigated catalytic model in longitudinal cohort in order to validate this approach. The authors compared SCR using antibody responses against the merozoite surface protein_1–19_ antigen measured in a prospective longitudinal cohort of children aged 0–11 years old to SCR calculated in a cross-sectional survey in a different group ranging from 0 to 90 years old. The comparison between the two approaches was done exclusively in children up to 11 years.

One of the objectives of the present study was to explore if the incorporation of some variations such as allowing age-varying to SCR or adding covariates in the reversible catalytic model could improve the precision or accuracy of exposure estimates using serological measurement of antibody responses to whole parasite extract. The availability of longitudinal data on a cohort whose age ranged from 0 to 90 years allowed the analysis of SCR and SRR values in different age group as studies have shown that antibody responses conversion and reversion varied with age and antigen [31–34].

A second objective of this study was to investigate the utility of *P. falciparum* crude schizonts extract which is a mixture of antigens, to monitor malaria transmission intensity and to detect temporal changes in malaria epidemiology in Dielmo, a Senegalese rural holoendemic area, using the age-specific reversible catalytic model described in previous studies [15, 16]. In Dielmo village, various malaria control interventions have been implemented since 2000, leading to significant reduction in malaria transmission and incidence [35]. Thirdly, the effects of potential covariates such as malaria control interventions and clinical malaria attacks on the SCR and SRR were investigated. Lastly, the use of cross-sectional data versus longitudinal data was also analysed and a comparison was performed between reversible catalytic model and alternative catalytic model in longitudinal data for assessing transmission evolution through SCR or SRR.

## Methods

### Study area and population

In this study, sera collected during five cross-sectional surveys in 2000, 2002, 2008, 2010 and 2012 from individuals living in Dielmo were used. Villagers are followed up longitudinally since 1990 as part of the Dielmo project [36, 37].

Different interventions were implemented based on recommendations from the National Malaria Control Programme. Treatments included the use of chloroquine in 2000, replaced by the association amodiaquine plus sulfadoxine–pyrimethamine from November 2003 to May 2006, followed by the introduction of artemisinin-based combination therapy (ACT) since June 2006. LLINs have been deployed since 2008. As a result, the village has experienced a dramatic decrease of the different malaria indicators monitored [35]. The incidence of clinical malaria dropped from 771 in 2000 to only 17 cases in 2012 [35]. Malaria transmission decreased considerably with EIR dropping from 500 infective bites/person/year in 2000 to 7.6 infective bites/person/year in 2012 [35]. For the longitudinal study, cross-sectional data were combined and only people who have at least two sera observations during the survey periods were considered in the longitudinal analysis. A total of 1147 sera obtained from 359 subjects aged 0–91 years old were used in the longitudinal analysis.

### Laboratory methods

*Plasmodium falciparum* crude schizonts extract obtained from a culture-adapted *P. falciparum* strain (Pf 07/03) isolated from an inhabitant of Dielmo, was used as antigens for serological investigations. Procedures of *P. falciparum* culture and crude extract schizonts (Sch 07/03) preparations have been described previously [38]. Antibody responses were assayed by ELISA, the cut-off value for positive antibody response was defined as previously described [38]. Briefly, antibody responses were considered positive if the optical density ratio (OD ratio = mean OD value of sample divided by mean OD value of naïve sera) was above two.

### Statistical analysis

#### Antibody levels comparison

Comparisons of OD ratio of antibody responses were assessed using Kruskal–Wallis rank test and this has been presented in a previous study [39]. Three age groups were studied: under 5, 5–15 years old and above 15 years old. Odds ratio for prevalence was determined using a simple logistic regression. Seroprevalence was estimated by the proportion of seropositive participants. Kruskal–Wallis rank test was used to compare ODs between surveys. Chi Square and Fischer exact tests were used to compare seroprevalences between age groups. All statistics were done using R software [40].

#### Reversible catalytic prevalence models for cross-sectional data

Seroconversion and seroreversion rates were calculated using the five observed cross-sectional data separately. At first instance, a previously described age specific reversible catalytic conversion model [15, 16, 18] was adjusted to the binary sero-immunological data (antibody responses to crude *P. falciparum* schizonts extract) to estimate seroconversion (λ) and seroreversion (ρ) rate, respectively. For each cross-sectional survey, seroprevalence has been calculated as described by Corran et al. [15] according to the following formula:

$$P\left( a \right) = \frac{\lambda }{\lambda + \rho }\left( {1 - \exp \left( { - \left( {\lambda + \rho } \right)a} \right)} \right)$$

λ: the annual seroconversion rate, ρ: the annual seroreversion rate, P(a): seroprevalence for a given age a.

Seroconversion and seroreversion rates represent respectively the force of exposure to malaria parasites over time relative to the intensity of infection in malaria endemic areas and the persistence of antibody response [16]. Secondly, an age specific variation in seroconversion rate (λ) and seroreversion rate (ρ) was investigated by adjusting an alternative catalytic model in which λ and ρ were allowed to change at time-point. Comparison of these models was performed using likelihood ratio test.

#### Estimation of seroprevalence and seroconversion rates with longitudinal data using reversible catalytic and alternative catalytic prevalence models

Age-specific antibody seroprevalence and seroconversion in longitudinal study were estimated by combining measurements obtained from all five cross sectional surveys. In order to select the best model for the serological longitudinal data, two approaches were used. First, a catalytic model and an alternative catalytic model were applied and λ (seroconversion rate) and ρ (seroreversion rate) were allowed to change at time-point without covariates. Second, these two models with covariates were applied to investigate factors that could influence both seroconversion (λ) and seroreversion (ρ) rates. In this study, covariates which were incorporated in the models were clinical malaria episode and use of bed nets. In these models seroconversion (λ) and seroreversion (ρ) rates were calculated respectively by:

$$\uplambda( {\text{t}}) = \uplambda_{0} *{ \exp }( \beta_{1} *{\text{clinical episode }} + \beta_{2} *{\text{use of mosquito bed nets}})$$

$$\uprho ({\text{t}}) = \uprho_{0} * \exp ( \beta^{\prime }_{1} *{\text{clinical}}\;{\text{episode }} + \beta^{\prime }_{2} *{\text{use}}\;{\text{of}}\;{\text{mosquito}}\;{\text{bed}}\;{\text{nets}})$$

where λ_0_(t) and ρ_0_(t) were annual conversion rate from seronegative to seropositive (without covariate effect) and annual reversion rate from seropositive to seronegative (without covariate effect), respectively. This model is similar to the one described by Sepulveda et al. [41], though the authors did not describe a model that allowed the SCR to vary by covariates. The regression coefficients (*β*_1_, *β*_2_, *β′*_1_, *β′*_2_)can be interpreted in terms of relative risk (RR) similarly to regression coefficient in the proportional hazards regression model of Cox [42]. Likelihood ratio test (LRT) and Wald test were used to assess the effect of covariates and to select the parsimonious model. Significant cut-off was fixed at 0.05 (p < 0.05). The goal here was to assess the effect of clinical malaria attacks and LLIN-based malaria control interventions on SCR. The reversible catalytic and alternative reversible catalytic models are detailed in Additional file 1.

#### Comparison between observed and predicted seroprevalence

The observed seroprevalence and predicted seroprevalence were compared using Log likelihood ratio test. Predicted seroprevalence curves and 95% confidence intervals were plotted and the corresponding λ was calculated for each cross-sectional period and both cross-sectional surveys combined. Seroprevalence using observed positive responses data were also represented. Wald test and likelihood ratio test (LRT) were used to test effects of covariates. All analyses were conducted using R software version 3.1.2 [40].

## Results

### Characteristics of the surveyed population

Table 1 presents epidemiological, demographical and clinical characteristics of the surveyed population. The age characteristics of the surveyed population were described in a previous study [39] Both total and mean number of clinical malaria episodes/person decreased over time. Prior to the introduction of LLINs in Dielmo in 2008, the maximum number of clinical malaria attacks varied between 14 and 17 episodes/person/year). Importantly, the maximum number of clinical malaria episodes/person decreased dramatically after the introduction of LLINs in the study site in 2008 [35] falling to 2 and 1 episode in 2010 and 2012, respectively. Table 2 describes the studied cohort; 35.1% of subjects were observed three times and 15.9% were observed five times during the study. Age medians were relatively similar for all screened times groups (Table 2), except the group that was observed five times during the survey period.

**Table 1:** Characteristics of survey participants

	2000 (N = 436)	2002 (N = 372)	2008 (N = 538)	2010 (N = 576)	2012 (N = 548)	p value
Sex						
Male	53.7 (234)	48.4 (180)	47.6 (256)	47.2 (272)	45.6 (250)	
Female	46.3 (202)	51.6 (192)	52.4 (282)	52.8 (304)	54.4 (298)	0.091
Clinical malaria episodes/person						
Min	0	0	0	0	0	
Mean	1.6	1.1	1.09	0.20	0.03	
Max	14	17	13	2	1

**Table 2:** Characteristics of the longitudinal cohort

Number of screened times during survey period	% of subjects (N = 359)	Age median
2	30.6 (110)	12.8
3	35.1 (126)	12.3
4	18.4 (66)	12.5
5	15.9 (57)	36

### Prevalence of antibody responses to Sch 07/03 amongst age groups

**Fig. 1:**
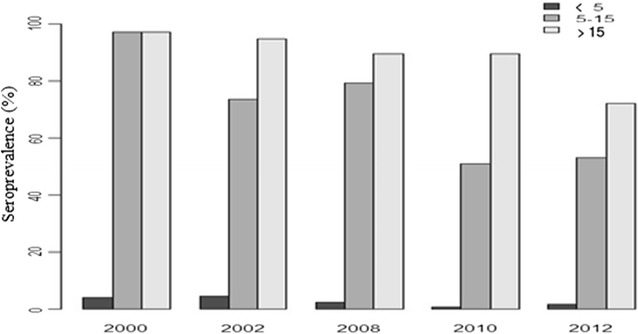
Seroprevalence to *P. falciparum* malaria schizonts sch07/03 with age groups by survey

Prevalence of antibodies responses against *P. falciparum* crude schizonts extract decreased significantly over surveys. Figure 1 shows the seroprevalence for each survey and by age group. Seroprevalence increased significantly with age per survey. Seroprevalence decreased over time during surveys in both children and adults. The reduction in seroprevalence was greater after the introduction of bet nets in the village in late 2008 for children and older children (5–15 years). For adults, reduction in seroprevalence was observed later after 2010. The proportion of seropositive subjects varied from 97% in 2000 to 72% in 2012. However, during the high transmission period in 2000, antibody responses were not different between older children (5–15 years) and adults whereas significant differences in seroprevalence were observed between these age groups in 2002, 2008, 2010 and 2012. Prevalence of antibodies was lowest among younger children (0–5 years). In this age group (0–5 years), the highest percentage was observed in 2002 (4.57%), significantly different to the two other age groups. Of note, in the youngest age group, the already low seroprevalence continued to decrease after the introduction of mosquito nets in 2008 despite a small increase in 2012 (Fig. 1).

In older children (5–15 years), seroprevalence decreased from 2000 to 2010 with a high rate of decay between 2008 and 2010 (79.27% of responders in 2008 and 50.98% in 2010). The rebound observed in 2012 in the young children (0–5 years) was also found in the older children group (5–15 years) (Fig. 1). For the adult group, seroprevalence decreased slightly between 2000 and 2012. Despite the decrease observed before and after 2008, the difference was not significant (p = 0.12).

### Analysis of seroconversion rate and seroprevalence curves by cross-sectional survey

**Fig. 2:**
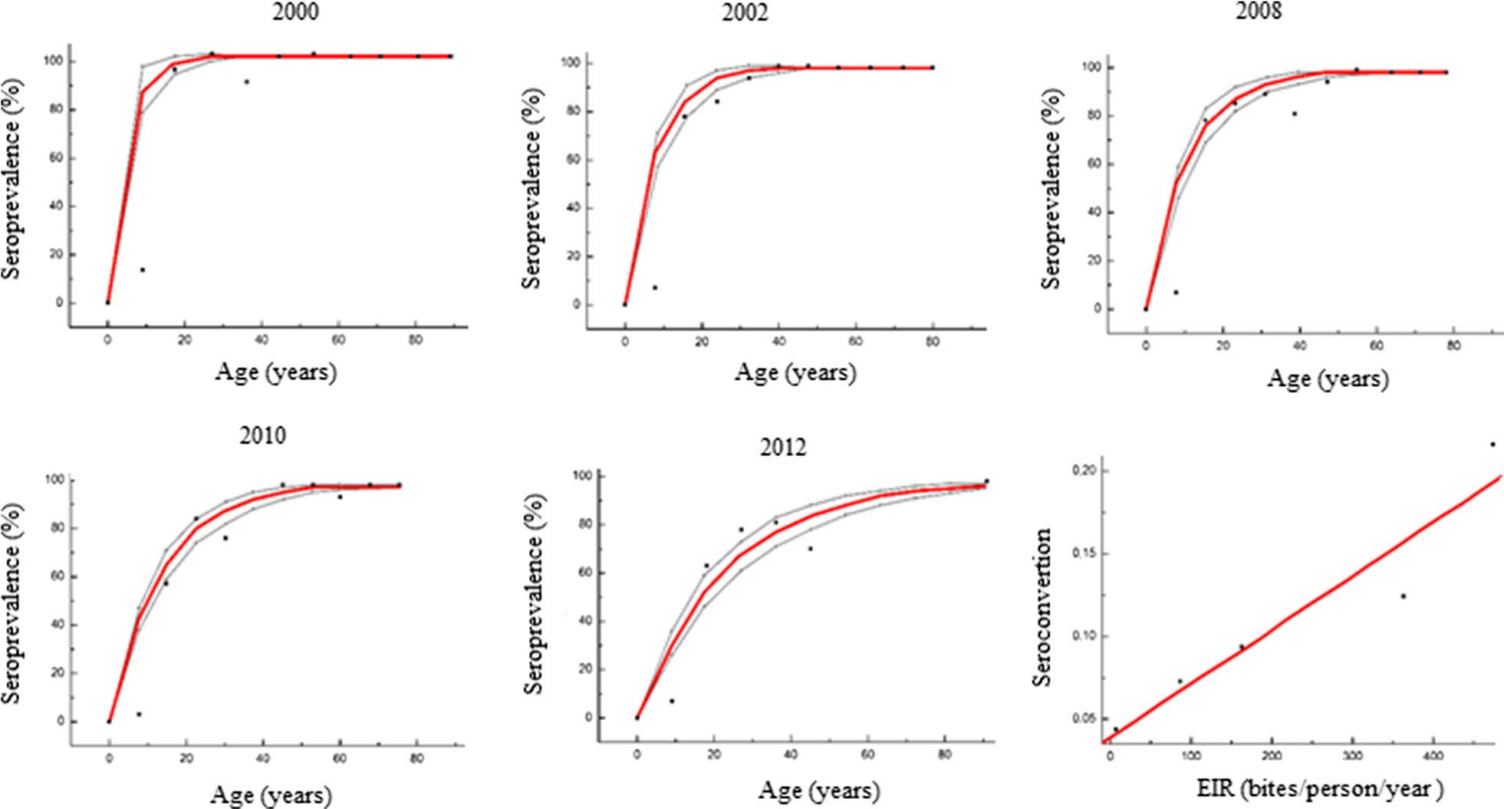
Fitting seroprevalence by reversible catalytic model against Pf Sh07/03 for each cross-sectional survey. Black dots represent the observed values, red lines indicate estimated seroconversion with the model and black broken lines indicate the 95% confidence interval (CI) with the likelihood ratio test. The sixth plot present the correlation between the seroconversion rate and the observed EIR given by entomologists

Seroprevalence was estimated using an age-dependent model, a reversible catalytic conversion model described previously [16]. However, the SCR was not fixed to one value, but could be modified [16, 25]. Figure 2 shows an increase in proportion of seropositivity with participant’s age. Comparison of the different predicted curves at different time points showed a difference in the rate of acquisition of positive responses with a decreased rate from 2000 to 2012. However, visual assessment of the plot suggested a poor fitting of the model for all surveyed time points. For instance, the observed seroprevalence was lower than the predicted one for the younger age group.

Significant difference of SCRs were observed between 2000 and 2008 (p = 0.024) and between 2002 and 2008 (0.011). SCRs were highest before the introduction of LLINs in 2008 with SCR values of 0.22 [95% CI (0.173–0.280)] and 0.126 [95% CI (0.099–0.159)] in 2000 and 2002 respectively compared to 0.094 [95% CI (0.079–0.112)] in 2008. In addition, a decrease of SCRs was observed after 2008 (Table 3). When an alternative catalytic model that included age specific variation for seroconversion rate was used, the best adjustment to the real data (LRT, p < 0.001) was observed as compared to the catalytic model with constant SCR and SRR.

**Table 3:** Seroconversion rates from catalytic models in cross-sectional surveys, their corresponding EIR [15] calculated from the serological measures and observed EIR given by entomologists

Surveys period	SCR (95% CI)	Corresponding EIR	Observed EIR
2000	0.22 (0.17–0.280)	518.57	482.6
2002	0.126 (0.099–0.159)	261.77	409.9
2008	0.094 (0.079–0.112)	174.35	155.3
2010	0.073 (0.062–0.086)	116.97	88.8
2012	0.042 (0.036–0.049)	32.28	7.6

### Model applicability: correlation between SCR and EIR

**Fig. 3:**
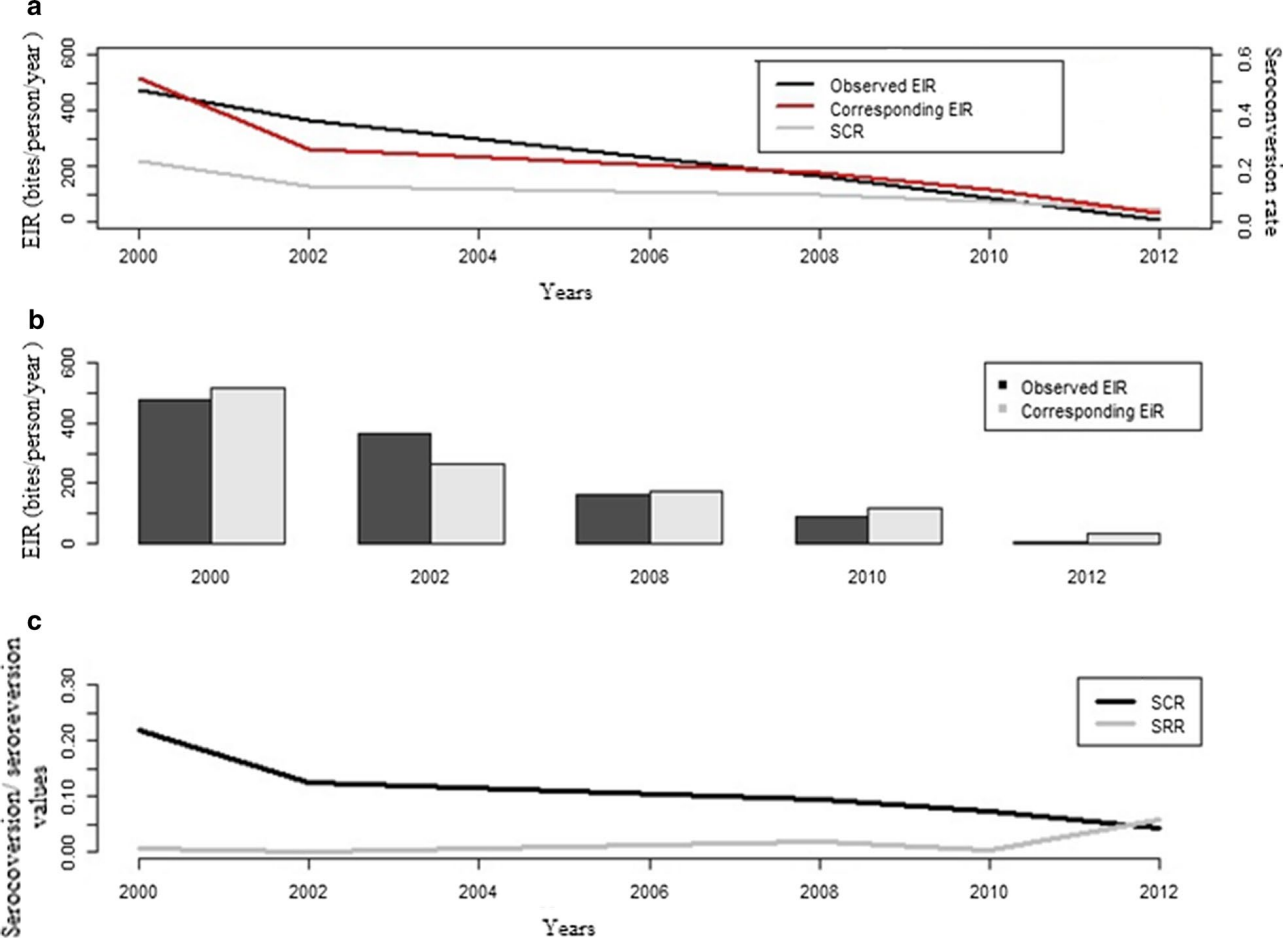
Trends of observed and estimated entomological inoculate rate (EIR), seroconversion rate (SCR) and seroreversion rate (SRR): **a** dynamic of observed entomological inoculate rate, EIR (black) calculated from parasitological measurements, seroconversion rate of anti-Pfsch07/03 and their corresponding entomological inoculate obtained by the catalytic model described previously [15], **b** comparison between observed and corresponding EIR obtained by the log–log calibration [15, 16], **c** evolution of SCR and SRR by years

A positive correlation was observed between EIR and SCR (λ) of antibodies against Pfsch07/03 (r-square = 0.905, p = 0.024). This trend was also shown in Fig. 3a, which presents the evolution of observed EIRs and corresponding EIRs calculated from serological measures using the method described in previous studies, respectively [15, 16].

The concomitant decrease of both EIR and SCR (λ) during survey period suggested that serological data could be used to assess the level of malaria transmission intensity. Figure 3b shows EIRs estimated from SCRs of antibody responses to *Pfsch07/03*—compared to observed EIRs measured with entomological methods in the study site (Dielmo) over the 5 years of surveys [35]. Estimated EIR showed a greater variability across survey periods. Despite slight difference between observed EIR and estimated EIRs from all period except for 2002, the corresponding EIRs closely followed up trend of observed EIRs over the survey periods (Fig. 3a).

Figure 3c describes dynamics of both SCR and SRR over the period. Dramatic reduction was observed in estimates seroconversion rates (SCR). In seroreversion rates (SRR), a small reduction was observed over time until 2010 where a slight increase was found.

### Analysis of seroconversion rate and seroprevalence curves in longitudinal survey

**Fig. 4:**
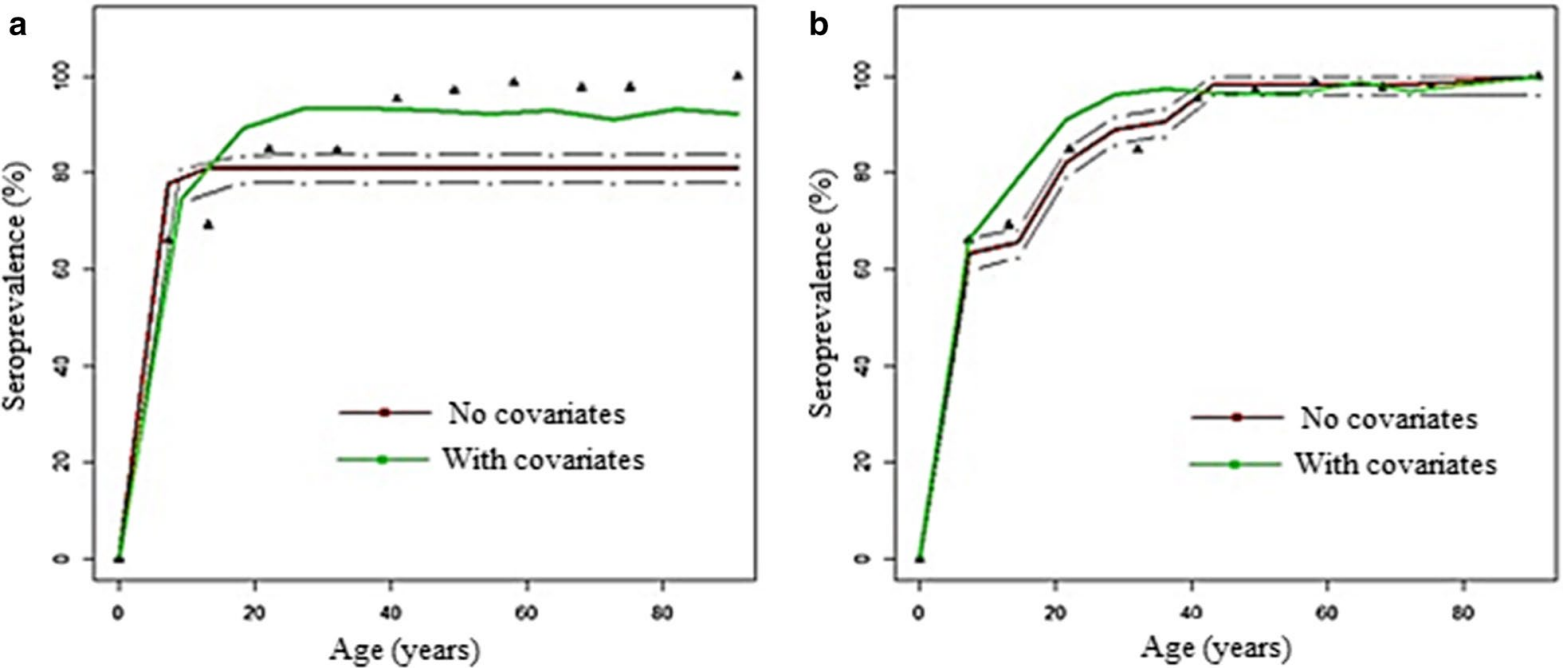
Fitting seroprevalence by reversible catalytic model (**a**) and alternative catalytic model (**b**) for anti-*Pf Sh07/03.* For all these figures, the red curves were the seroprevalence obtained from models without covariates and their 95% likelihood ratio test confidence intervals and the green ones these from models according to the clinical attacks and the LLINs usage. The triangle points were the seroprevalences calculated among the observed antibodies response

Seroprevalence results from catalytic model applied to longitudinal data are shown in Fig. 4a. A poor fitting of age-seroprevalence of anti-*Pfsch0703* was observed. Seroprevalence in adults group was under-estimated compared to younger age groups, which seemed to be over-estimated. Seroconversion and seroreversion rates estimated from the catalytic model was 0.284 and 0.066, respectively (Table 4).

**Table 4:** Estimation of seroconversion and seroreversion rates from longitudinal and cross-sectional studies

Study	Seroconversion (95% CI)	Seroreversion (95% CI)
Longitudinal survey		
Without covariates	0.284 (0.230–0.351)	0.066 (0.051–0.085)
With covariates	0.215 (0.171–0.269)	0.041 (0.030 - 0.055)
Cross-sectional surveys		
2000	0.220 (0.173–0.280)	0.006 (0.00–0.514)
2002	0.126 (0.099–0.159)	0.001 (0.00–0.02)
2008	0.094 (0.079–0.112)	0.017 (0.007–0.047)
2010	0.073 (0.059–0.091)	0.003 (0.00–0.024)
2012	0.042 (0.036–0.049)	0.057 (0.026–0.125)

Slight variation of SCRs and SRRs were observed when covariates such as clinical malaria attacks and LLINs usage were taken into account in the catalytic model in which SCR and SRR were estimated respectively at 0.284 (0.230–0.351) and 0.041 (0.030–0.055). The LRT showed a significant difference between these models in favor of the model with covariates (p < 0.001).

Figure 4b presents results of the alternative catalytic model in which age-varying SCR and SRR are allowed. The LRT showed that the alternative model was significantly better than the catalytic model in which SCR and SRR were constant (*X* ^2^ = 25.67, p < 0.001). Current SCR (2012) using all sample surveys was calculated and found to be equal to 0.056 [95% CI (0.04–0.11)] from the alternative catalytic model and the corresponding SRR was 0.01 [95% CI (0.0007–0.08)].

### Comparison of SCR and SRR from longitudinal study *versus* cross-sectional surveys

Table 4 shows comparison of SCR and SRR estimated from longitudinal surveys to those from the five cross-sectional surveys by both catalytic models with and without covariates. SCRs from longitudinal approach [SCR (without covariates) = 0.284 (0.230–0.351) and SCR (with covariates) = 0.215 (0.171, 0.269)] were almost equal to the SCR (0.220 (0.173, 0.280) from the cross-sectional survey in 2000. For other cross-sectional surveys (2002, 2008, 2010 and 2012), there were a high variability between SCRs from cross-sectional and longitudinal surveys. In contrast, for the alternative catalytic model with covariates current SCR estimated to 0.046 (0.003–0.052) was comparable to SCR value of 0.042 (0.036–0.049) from the cross-sectional survey in 2012 (Fig. 4b).

### Effects of clinical malaria attack and malaria control interventions on seroconversion

To investigate respectively the impact of LLINs usage and clinical malaria episodes on SCR and SRR, catalytic age reversion model with covariates has been applied on data combined from all surveys. Table 5 summarizes the estimation of these parameters. The mosquito nets intervention reduced the seroconversion rate, nevertheless it increased the seroreversion rate. A significant association was found between clinical episodes and SCR with a p value < 0.001.

**Table 5:** Estimation of parameters [respectively the relative risk (RR)] influencing seroconversion and seroreversion rates from longitudinal studies

Covariates	SCR			SRR		
	Effect	RR	p value	Effect	RR	p value
Clinical episodes	0.08	0.092	< 0.001	0.24	0.786	< 0.001
Mosquito nets	− 2.57	0.076	< 0.001	0.010	1.01	< 0.001

## Discussion

In malaria pre-elimination context with reduced clinical episodes and transmission intensity due to implemented strategies against the disease, standard assessment tools lack of sensitivity and more sensitive tools are necessary to accurately evaluate both the level of exposure and the impact of malaria control interventions. These tools can also help to detect resurgence in transmission intensity. In recent years, several studies described the estimation of malaria transmission using model based on the seroprevalence of antibody responses against recombinant blood stage antigens, such as MSP1, MSP2, AMA-1, MSP-1_19, GLURP [15–18, 27–30, 43, 44] and sporozoite stage antigens of *Plasmodium* [45]. These serological markers could be potentially sensitive, especially in areas of low transmission [29] as antibody responses, in particular to blood stage antigens, have been shown to persist for several years after transmission has ceased [46–49]. Thus, antibody responses against blood stage antigens could be detected even if transmission is lower. However, the use of specific single antigen might induce a lack of sensitivity and lead to a significant underestimation of the immune response to *P. falciparum* infection. The variation in individual immune reactivity as well as the different duration of antibody responses against different antigens [34, 50] and antigenic polymorphism may affect serological responses, thus the results obtained with specific recombinant antigens [21–23, 51]. As an example, Ondigo et al. showed that antibody half-life may be short, intermediate or long depending on age and antigen and concluded for a need to associate antigen with similar half-life to improve precision and accuracy of malaria exposure estimates [52]. Recently, Helb et al., in a study aimed at improving tools to reliably measure *P. falciparum* exposure, observed that *P. falciparum*-specific antibody responses differ by antigen and, in contrast with Ondigo et al., concluded to the need of selecting and combining different antigens with different kinetics for improving estimates of *P. falciparum* exposure [53].

These combinations have been demonstrated to improve the accuracy and the precision of malaria infection exposure. However the technologies used (multiplex, microarrays or cytometry) are quite expensive and the required equipment is not always available (or only in very few, high-level laboratories) in the countries where monitoring of malaria infection is needed. In a country with limited resources, there is probably a space for the use of less sophisticated tools, such as ELISA assays, and based on the results of this study, the use of crude extract could be a good choice. In fact, crude extract containing a plurality of antigens can overcome the constraints associated to the choice of specific antigens and give a better sensitivity, allowing detection of *P. falciparum* exposure in areas of low-level transmission [22, 24].

In this current study, a crude schizont extract of *PfSch07/03* was used in ELISA to examine the dynamic of antibody responses in individuals living in Dielmo (Senegal), an area where malaria transmission has substantially decreased [35]. An estimation transmission model based on prevalence of antibody responses against *PfSch07*/03 was applied to assess the applicability of this model in the study area.

Results showed a significant increase both in level and prevalence of antibody responses with age within surveys. These observations confirmed the general trend that anti-malaria immune responses varied with age in endemic areas [38]. For all age groups, comparisons of both OD magnitude and seropositivity prevalence showed that antibody level and seroprevalence were generally significantly higher in 2000 and decreased over time to 2012. The decreased of antibody responses correlated with the decrease of transmission level induced by control interventions deployed in Dielmo [35]. These results confirmed the high sensitivity of serological responses to detect changes in exposure to malaria infection [15, 16].

Maximum likelihood method from age-specific seroprevalence reversible catalytic model was used to estimate seroconversion rate (SCR). SCR estimates were shown to correlate closely with EIR, the gold standard measurement of malaria transmission. The result was consistent with previous studies that have shown good correlation between EIR and SCR in low transmission areas [15]. Importantly, heterogeneity of SCR by time-point survey corresponded to that of transmission in the study site. Indeed, the study locality has for long been a holoendemic malaria area in which transmission has varied from 142.5 (in 1990) to 482 (in 2000) infective bites/person/year [35]. A recent study in this locality has shown that both transmission level and malaria attacks dropped since the introduction of LLINs in 2008 combined with ACT [35]. The data showed the recent change about the level of transmission in Dielmo. Some cautiousness should be considered on the interpretation of the relationship between SCR and EIR. The variability of these measures should be taken into account in order to avoid misinterpretation. However, estimating the level of malaria transmission with the serological tool is relatively simple and could have an interest in the assessment of control interventions all the more so the antibodies response remain detectable for years.

SCRs obtained by combining all sectional data were statistically different from those obtained with time-point survey. In cross-sectional studies, SCRs gave precise information in the level of transmission and detected previous changes in malaria exposure. This difference could be explained by the heterogeneity in the transmission in Dielmo during these periods [35] and by antibody responses variability [38]. In contrast, the study of Arnold et al. [30] found a similarity between the prospective estimate of the seroconversion rate from the longitudinal data and that estimated from the cross-sectional survey using data that covered the same period (cross-sectional sample in 2012). These contrasting results could be explained partly by the difference of samples in these two studies. Indeed the samples used by Arnold et al. [30] included only children aged 0–11 years old. Another explanation could be the dramatic reduction in malaria transmission over the 12 years study period, while in the study by Arnold et al. [30]. the transmission was constant over the study period.

The observation that SCR averaged over the entire period in the longitudinal analysis differed from the weighted average of the stratum-specific SCRs could be the consequence of the large change in SCR values over the study period. In view of the results in the present study. It seemed that the use for longitudinal data of catalytic models with constant SCR and SRR is not suitable in areas with rapid decline in malaria transmission. In these areas models allowed for time-varying SCR and SRR could be more adapted for longitudinal data in order to take into account the changes in SCRs following the reduction of malaria infection exposure.

Investigation of factors which might affect the SCR and SRR showed that both clinical episodes and the use of mosquito nets had significant impact on these parameters. In agreement with previous studies [18] showing that the incidence of the disease has a significant influence on the estimation of SCR, clinical episodes were positively associated with the SCR of antibody responses to *PfSch07/03*. Similarly, the use of mosquito nets reduced the risk of antibodies jumping from seronegative to seropositive state. This is consistent with previous studies that have shown significant decrease in the level of immunity in the locality after the introduction of LLINs [38].

Results of LRT and Wald test were in favor of the alternative model suggesting that the previously described catalytic model [15, 16] was restrictive both in cross-sectional and in longitudinal studies in Dielmo. However, this model gave some interesting results, among which, the correlation between corresponding EIRs obtained by serological data and observed EIR (entomological measurement). These results suggested the applicability of the catalytic model to serological data. However, as described by Yman et al. [54] the use of catalytic model has some weakness such as the conversion of serological data to binary outcomes through a threshold model and the choice of this threshold. Furthermore, dichotomizing serological measures (seronegative *vs* seropositive) may lead to a loss of information due to the high range of serological values of the seropositive group. Several authors proposed alternative models using continuous serological data, as the density model [41, 42] or the antibody acquisition model [54]. Interestingly Pothin et al. have shown that while seroconversion rate from the catalytic model and exposure rate from the density model measure different quantities, a high correlation has been observed between these two measures [43]. This consolidates the use of the catalytic model in this study. However, in the antibody acquisition model, Yman et al. found that in settings of moderate and intense transmission, this model based on continuous data gave better precision and accuracy compared to catalytic models [54], even if the authors agreed that this approach has some disadvantages in particular if the data are not log-normally distributed [54]. Arnold et al. proposed the use of quantitative antibody levels as a valuable method to measure changes in transmission or difference in exposure for pathogens that elicit a transient antibody response or for monitoring populations with very high- or very low transmission [55]. Indeed, in previous studies on the population of the present study, in parallel to seroprevalence estimations, quantitative antibody levels were measured in different age groups and compared between two very different periods of malaria transmission [38]. The results showed that the levels of antibody responses added complementary and valuable information to seroprevalence values.

Comparisons of the different methods with the same data could be interesting to envisage.

## Conclusions

The present study underlines the capacity of serological responses using crude extracts of *P. falciparum* to detect changes in malaria transmission and suggests to take into account malaria control interventions in the estimation of the serological measurements of malaria transmission. Further investigations comparing SCRs obtained from recombinant antigens and especially vaccine candidates antigens to those obtained from crude extracts would provide the basis for the choice and validation of a reference serological marker to assess the level of malaria transmission in relation to changing epidemiology. Additionally, estimations of seroconversion rates should take into account the heterogeneity of the transmission within a study period and the methodology used for these estimations should be adapted to the epidemiology context.

## Additional file

**Additional file 1.** This supporting information contains statistical details. It includes information about models used in the analysis and the estimates methodologies.
