# Supplementary material for: Modelling dynamic change of malaria transmission in holoendemic setting (Dielmo, Senegal) using longitudinal measures of antibody prevalence to Plasmodium falciparum crude schizonts extract
Source: Malar J. 2017 Oct 11;16:409. doi: 10.1186/s12936-017-2052-0 (PMC5637097; doi:10.1186/s12936-017-2052-0)
Supplement: Supplementary file 1 — Additional file 1. This supporting information contains statistical details. It includes information about models used in the analysis and the estimates methodologies. [file 12936_2017_2052_MOESM1_ESM.docx]

**Modelling dynamic change of malaria transmission in holoendemic setting (Dielmo, Senegal) using longitudinal and Cross-sectional measures of antibody prevalence to *P. falciparum* crude schizonts extract**

Niass Oumy^1,2^ (oumyniass@gmail.com), Philippe Saint-Pierre^3^ (philippe.saint_pierre@math.univ-toulouse.fr), Faye Michel Matar ^1^(mfaye@pasteur.sn), Diop Fode^1^([fdiop@pasteur.sn](mailto:fdiop@pasteur.sn)), Niang Makhtar^1^([mniang@pasteur.sn](mailto:mniang@pasteur.sn)), Diouf Babacar^1^([bdiouf@pasteur.sn](mailto:bdiouf@pasteur.sn)), Faye Joseph ([jfaye@pasteur.sn](mailto:jfaye@pasteur.sn)), Diagne Nafissatou ^4^ (nafissatouDiagne.ird.fr),. Sokhna Cheikh ^4^ (Cheikh.Sokhna@ird.fr), Trape Jean-François ^4^ ([jean-francois.trape@ird.fr](mailto:jean-francois.trape@ird.fr), Perraut Ronald^1^ ([perraut@pasteur.sn](mailto:perraut@pasteur.sn)), Tall Adama^5^ ([tall@pasteur.sn](mailto:tall@pasteur.sn)), Diongue Abdou Kâ ^2^ (abdou.diongue@ugb.edu.sn) and Toure Balde Aïssatou ^1^ ([atoure@pasteur.sn](mailto:atoure@pasteur.sn))

This document gives supplement informations for data analysis and statistical methods used to the paper. In the first section we describe the historic of the catalytic model in a longitudinal case, methodologies to incorporate covariates in the model and give technics to estimate the parameters of the model. In the second section, we define a variant of this model called the alternative catalytic model. In the last section we detail the catalytic model for cross-sectional surveys.

1. **The reversible catalytic model for longitudinal cohort**

This model was firstly proposed in 1976 by Bekessy et al.[[1](#_ENREF_1)]. They assume that, in a given period and in a given population in which for an infection, negatives become positives and positives become negatives, the dynamic of the infection follows a Markov process in character, continuous in time, and with two states only. State 1 is called the "negative", i.e., the infection-negative state, and state 2 the "positive" one. This model supposed that the process is homogeneous, which means that the transition probabilities between two time periods t and t+1 have not depend to the information before t. With these hypothesis transition probabilities are stationary and the relationships between transition rates and transition probabilities is known. Thus, after resolution of the Kolmogorov differential equations, the probability of infection and of recovery respectively in a period t is  [[1](#_ENREF_1)]:

$$P \left( t \right)= \frac{\lambda}{\lambda+ \rho} \left( 1-\exp\left( -\left( \lambda+ \rho\right)t \right) \right)$$

(1)

$$P \left( t \right)= \frac{\rho}{\lambda+ \rho} \left( 1-\exp\left( -\left( \lambda+ \rho\right)t \right) \right)$$

Where $\lambda$ and $\rho$ are the transition intensities commonly called respectively incidence rate and recovery rate. In serological context $\lambda$ was named seroconversion rate and $\rho$ seroreversion rate. Parameters of the models can be estimated by using maximum likelihood approach.

Let the states be numbered 1 and 2. Suppose a sample constituted by $n$ subjects denoted by $h (h=1,2, \ldots)$ whose are observed over the time interval (0, τ]. The h-th subject moves $n_{h}$ times. The observed data for subject $h$ consist of ${0=t}_{0}^{h}< t_{1}^{h} <\ldots< t_{n_{h}}^{h}$ = τ which are the successive follow-up times. Let $n_{ijl}^{h}$ denote the number of time that the subject $h$ was observed to be in state $i$ and then observed to be in state $j$ at the next inspection. The likelihood function can be calculated to

$$L( \vartheta)=\prod_{h=1}^{n} \left\{ \prod_{l=1}^{n_{n}} \prod_{i=1}^{2}\prod_{j=1}^{2} \left( P_{ij}(s_{l}^{h}; \vartheta) \right)^{n_{ijl}^{h}} \right\}$$

Where $\vartheta=(\lambda; \rho)$ and$s_{l}^{h}=(t_{l}^{h}- t_{l-1}^{h})$. Parameters ($\vartheta=(\lambda; \rho))$ estimation can be carried by severous methods. Here we used the quasi-Newton algorithm to find maximum likelihood parameter’s estimates. Confidence intervals (CI) standard deviation have been calculated by using the bootstrap approach with 10000 replication.

- **Incorporation of covariates in the model.**

In many healthy studies, for each subject some covariates are measured and it is interesting to assess the relationship between these covariates and the seroconversion rate $\lambda$ and seroreversion rate $\rho$ in the Markov model. The model can be extended in a straightforward way to allow for regression modelling of $\lambda$ and $\rho$. We assume the proportional intensities regression models, then the seroconversion rate can be expressed as

$$\lambda= ʎ_{0}exp\left( \sum_{k=1}^{p} \beta_{k}Z_{k} \right)$$

Where $Z=\left( Z_{k} \right)_{k=1, 2, \ldots, p}$ is a p-dimensional vector of covariates, $\beta$ a vector of p regression coefficients relating the influence of Z to the seroconversion rate $\lambda$ (respectively to the seroreversion rate) and $ʎ_{0}$ represents the baseline seroconversion rate if any covariates have not impacted to the antibodies conversion. Here Z is a matrix with two covariates (clinical episode and the use of mosquito net), in this case seroconversion rate (ʎ) and seroreversion (ρ) rate have been calculated respectively by:

ʎ = $ʎ_{0}$*exp ($\beta_{1}$ *clinical episode + $\beta_{2}$*mosquito net)

ρ = $\rho_{0}$* exp (${\beta'}_{1}$ *clinical episode + ${\beta'}_{2}$*mosquito net)

The regression coefficients ($\beta_{1}$, $\beta_{2}$ ${\beta'}_{1},$ ${\beta'}_{2}$) can be interpreted in terms of relative risk similarly to regression coefficient in the proportional hazards regression model of Cox [[2](#_ENREF_2)].

1. **The alternative reversible catalytic model for longitudinal cohort**

The assumption of constancy of the transition rates is often so restrictive, mainly if we study evolution of the process during a long time. It is important to extend the basic model by allowing transition rate to change at some time point in the time interval. This model has been described previously by Alioum and Commenges [[3](#_ENREF_3)]. Let us divide the time axis into intervals


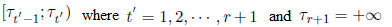
. We assume that seroconversion and seroreversion rates are constant in each interval.


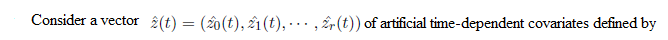


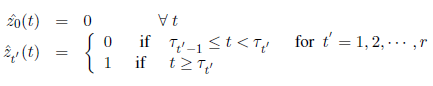


The seroconversion respectively the seroreversion rate vary with time as step-function defined on the pre-specification intervals:


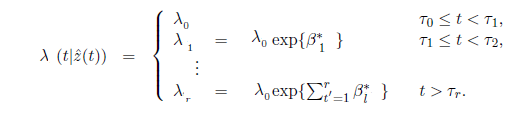


The seroreversion was writing at the same format. The parameters of this model are the baseline seroconversion and baseline seroreversion rates${( ʎ}_{0}, \rho_{0}$ ), and the vector of regression coefficients associated with the artificial time-dependent covariates. We use the likelihood method for estimating these parameters. For more information about this model, we refer the lecturers to the paper of Alioum and Commenges [[3](#_ENREF_3)].

**The reversible catalytic model for cross-sectional data.**

In the case of cross-sectional data, for each subject we disposed only the information about his current state for a given time. Indeed subjects have been observed only at one time point thus information about seroconversion and seroreversion are not directly know. Authors in previous studies used catalytic reversible model described in the previous section to model age- specific prevalence of antibody responses [[4](#_ENREF_4),[5](#_ENREF_5),[6](#_ENREF_6),[7](#_ENREF_7)]. In this model there is one probability which is the probability of positivity. This probability is given by

$P \left( k \right)= \frac{\lambda}{\lambda+ \rho} \left( 1-\exp\left( -\left( \lambda+ \rho\right)k \right) \right)$ (2)

It is the same equation to (1), except that here instead of using the subject-time in a period of observation t, we modele the prevalence according to the age k.

We adjust age-specific reversible catalytic model to five cross-sectional data separately. Estimates of $\lambda$ and $\rho$ were obtained by using the maximum likelihood estimation approach. For cross-sectional data we also fit a model in which we allow age-specific varying to $\lambda$ and to $\rho$.

**Reference**

**1. Bekessy A ML, Storey J (1976) Estimation of incidence and recovery rates of plasmodium falciparum parasitemia from longitudinal data. Bull World Health Organ 54: 685-693.**

**2. Cox DR (1972) Regression models and life tables (with discussion): J. R. Stat. Soc. B. 187-220 p.**

**3. Alioum A, Commenges, D. (2001) Mkvpci : a computer program for markov models with piecewise constant intensities and covariates. Comput Methods Programs Biomed 64: 109-119.**

**4. Corran P, Coleman P, Riley E, Drakeley C (2007) Serology: a robust indicator of malaria transmission intensity? Trends Parasitol 23: 575-582.**

**5. Bousema T, Youssef RM, Cook J, Cox J, Alegana VA, et al. (2010) Serologic markers for detecting malaria in areas of low endemicity, Somalia, 2008. Emerg Infect Dis 16: 392-399.**

**6. Stewart L, Gosling R, Griffin J, Gesase S, Campo J, et al. (2009) Rapid assessment of malaria transmission using age-specific sero-conversion rates. PLoS One 4: e6083.**

**7. Drakeley CJ, Corran PH, Coleman PG, Tongren JE, McDonald SL, et al. (2005) Estimating medium- and long-term trends in malaria transmission by using serological markers of malaria exposure. Proc Natl Acad Sci U S A 102: 5108-5113.**
